# RAF inhibitor re-challenge therapy in BRAF-aberrant pan-cancers: the RE-RAFFLE study

**DOI:** 10.1186/s12943-024-01982-4

**Published:** 2024-03-26

**Authors:** Blessie Elizabeth Nelson, Jason Roszik, Jibran Ahmed, Carmelia Maria Noia Barretto, Mirella Nardo, Erick Campbell, Amber M Johnson, Sarina A. Piha-Paul, Isabella C. Glitza Oliva, Shiao-Pei Weathers, Maria Cabanillas, Milind Javle, Funda Meric-Bernstam, Vivek Subbiah

**Affiliations:** 1 Department of Investigational Cancer Therapeutics, University of Texas MD Anderson Cancer Center, Houston, TX USA; 2 Department of Melanoma Medical Oncology, University of Texas MD Anderson Cancer Center, Houston, TX USA; 3 Department of Neuro-Oncology, University of Texas MD Anderson Cancer Center, Houston, TX USA; 4 Department of Endocrinology, University of Texas MD Anderson Cancer Center, Houston, TX USA; 5 Department of Gastrointestinal Medical Oncology, University of Texas MD Anderson Cancer Center, Houston, TX USA; 6 Early-Phase Drug Development, Sarah Cannon Research Institute, Nashville, TN USA

**Keywords:** Rechallenge, RAF pathway, BRAF inhibitors, BRAF-altered solid tumors

## Abstract

Previous studies have shown the clinical benefit of rechallenging the *RAF* pathway in melanoma patients previously treated with BRAF inhibitors. 44 patients with multiple tumors harboring *RAF* alterations were rechallenged with a second RAF inhibitor, either as monotherapy or in combination with other therapies, after prior therapy with a first RAF inhibitor. This retrospective observational study results showed that rechallenging with RAFi(s) led to an overall response rate of 18.1% [PR in thyroid (1 anaplastic; 3 papillary), 1 ovarian, 2 melanoma, 1 cholangiocarcinoma, and 1 anaplastic astrocytoma]. The clinical benefit rate was 54.5%; more than 30% of patients had durable responses with PR and SD lasting > 6 months. The median progression-free survival on therapy with second RAF inhibitor in the rechallenge setting either as monotherapy or combination was shorter at 2.7 months (0.9-30.1 m) compared to 8.6 months (6.5-11.5 m) with RAF-1i. However, the median PFS with RAF-2i responders (PFS-2) improved at 12.8 months compared to 11.4 months with RAF-1i responders. The median OS from retreatment with RAF-2i was 15.5 months (11.1-30.8 m). Further prospective studies are needed to validate these results and expand targeted therapy options for *RAF*-aberrant cancers.

**Supplementary Information:**

The online version contains supplementary material available at 10.1186/s12943-024-01982-4.

## Introduction

*BRAF* (v-RAF murine sarcoma viral oncogene homolog B1) operates as a serine/threonine protein kinase in the *MAPK* (mitogen-activated protein kinase) signaling pathway and it phosphorylates and activates downstream *MEK1* and *MEK2* (mitogen-activated protein kinase kinase 1 and 2) and their downstream *ERK1* and *ERK2* (extracellular signal-regulated protein kinases 1 and 2) proteins. *BRAF* is activated by the upstream *RAS* (rat sarcoma) GTPase (guanosine-nucleotide-binding protein). This signal transduction is involved in directing cell differentiation, growth, and survival. *BRAF* protein has three highly conserved domains from the point of evolution, CR1 (RAS-binding domain), CR2 (serine/threonine rich domain), and CR3 (kinase domain) that are involved in the activation of *BRAF* [1]. This occurs through the binding of active GTP-bound *RAS* to the N-terminal of CR1 and phosphorylation of CR2 which removes autoinhibition with subsequent activation of the catalytic kinase domain.

Oncogenic *BRAF* driver alterations, most commonly the class I *V600E*, are present variably across different cancers but most commonly in cutaneous melanoma and anaplastic thyroid cancer and a smaller percentage in colon cancer, non-small cell lung cancers, and several other tumor types. Other pathogenic alterations such as amplifications and fusions are less prevalent across tumors [2]. *BRAF* alterations are categorized as either Class 1, which includes *V600*, and these are activating *RAS*-independent monomers; Class II, activating *RAS*-independent dimers; And class III, which are active *RAS*-dependent alterations [3]. 

Over the years several BRAF V600 inhibitors (alone or in combination with MEK inhibitors) have been developed and have shown objective responses and survival benefits across several cancers and leading to tumor-specific, tissue-agnostic, and companion diagnostic FDA (Food and Drug Administration) approvals [4]. 

Although it is known that intratumor heterogeneity can lead to variable responses, however unfortunately, clonal evolution and development of acquired resistance mechanisms can occur during treatment which limits the clinical efficacy of these agents. Mechanisms for acquired resistance can be diverse such as secondary *MAPK* mutations, over-expression of tyrosine kinase receptor, activation of alternative resistance pathways such *as PI3K/AKT* (phosphoinositide 3-kinase/Ak strain transforming), or new alterations in *MAPK*-independent pathways and even histological transformation [5]. In melanoma, for example, resistance to treatment commonly develops due to mutations in *BRAF* splice variants and amplifications, and *NRAS* (neuroblastoma RAS viral oncogene homolog) mutations among other mechanisms [6]. Thus, interest has also grown over the development of the next generation of therapies targeting *BRAF* resistance mechanisms and class II and III alterations to continue to target the alterations in tumors.

Rechallenging patients with a BRAF inhibitor refers to retreatment for those who initially experienced clinical benefit to a BRAF treatment but later developed disease progression especially in the metastatic setting while being treated with these inhibitors and subsequently underwent rechallenge with RAF targeting therapy. It should be noted that period off therapy and timing of rechallenge are important considerations when rechallenging patients [7]. 

Studies have explored temporary or reversible resistance to BRAF inhibitors and a ‘plastic’ tumor phenotype which may revert upon removal of BRAF inhibition, for example, changes observed in the tumor microenvironment (TME) by matrix remodeling and therapeutic escape in melanoma cells [8]. Over time, the diminishing impact of these resistance changes in tumors may create an opportunity to target the BRAF mutation again with the ultimate goal of deriving clinical benefit.

The aim of this study was to examine the activity of RAF inhibitors (RAFi) among patients with BRAF-aberrant solid tumors, who underwent rechallenge with RAF pathway-directed treatment in the context of early-phase clinical trials.

## Methods

This was a retrospective study conducted in a single institution approved by The University of Texas MD Anderson Cancer Center’s Institutional Review Board. All patients treated as part of the clinical trial provided written informed consent. The demographic, clinical, and histopathologic data of patients were retrospectively collected and analyzed. Trial designs, schedules, and assessments varied among the clinical trials involved.

### Statistical analysis

Demographic and clinical characteristics were analyzed using descriptive statistics. PFS was defined as the time from the first day of cycle 1 to the date of progression or death, whichever came first. Patients who were alive and progression-free at the last clinical follow-up were censored at the date of the last clinical follow-up. OS was defined as the time from the first day of cycle 1 to death from any cause. Patients alive at the last follow-up were censored at the date of the last contact. Survival (PFS and OS) were analyzed using the Kaplan-Meier method from the time of trial participation and included median survivals (with 95% CIs). HRs and corresponding CIs and P values were computed using a Cox proportional hazards regression analysis. Clopper-Pearson exact binomial CIs were provided for estimates of proportions. Survival differences between treatment cohorts were assessed through the log-rank test with univariate analysis. All tests were 2-sided, and P values < 0.05 were considered statistically significant. All statistical analyses were performed using R software, v3.6.0.

### Genomic analysis

Archived tumor specimens were analyzed at institutional Clinical Laboratory Improvement Amendments (CLIA)-certified laboratories for next-generation sequencing data. Data was reviewed using other platforms such as NeoGenomics, Aliso Viejo, CA, USA; Guardant360; Guardant Health, Redwood City, CA; and Foundation Medicine, Cambridge, MA, USA.

## Results

### Treatment with RAF-1 inhibitor

#### Patient characteristics

Between January 2010 and November 2022, 44 patients with *BRAF* aberrated advanced solid tumors who received the first RAFi (RAF-1i) as monotherapy or in combination and were rechallenged with a second RAFi (RAF-2i) with or without other therapies at the Clinical Center for Targeted Therapy, University of Texas MD Anderson Cancer were identified for study analysis. Patients received therapy either as standard of care or as part of a clinical trial. RAF-1i and RAF-2i included class I, pan-RAF or dimer-selective RAF inhibitors. The Institutional Review Board independently reviewed and approved each clinical trial in which patients presented within this analysis were enrolled. The patients provided written informed consent before treatment with investigational therapy. All procedures conformed with the ethical standards of the institutional research committee and with the Declaration of Helsinki. Patient and disease characteristics before rechallenging are shown in Table S1.

The median age of trial participants was 54.5 years (25 years – 76 years) while in gender distribution, male participation was predominant (*n* = 26; 59%) compared to female participation (*n* = 18; 41%). The majority of the patients were of Caucasian ethnicity (*n* = 36; 82%) followed by Hispanic (*n* = 6; 14%) and African-American (*n* = 1; 2%) and Asian ethnicities (*n* = 1; 2%) Tumor types and histologies included cutaneous melanoma (*n* = 16; 36%), colorectal carcinoma (CRC) (*n* = 10; 23%), thyroid cancers with papillary thyroid histology (*n* = 3; 7%), anaplastic histology (*n* = 1; 2%), Central Nervous System (CNS) tumors including glioblastoma (*n* = 2; 5%), pleomorphic xanthoastrocytoma (*n* = 1; 2%), anaplastic astrocytoma (*n* = 1; 2%). Other tumor types included cholangiocarcinoma (*n* = 3; 7%); pancreatic ductal adenocarcinoma (PDAC) (*n* = 2; 5%), ovarian serous carcinoma (*n* = 2; 5%), non-small lung cancer of adenocarcinoma histology (*n* = 1; 2%), triple negative breast cancer (*n* = 1; 2%) and neuroendocrine carcinoma (*n* = 1; 2%). All patients had locally advanced, recurrent, or metastatic disease before RAF-1i therapy. 22% of patients had 1–2 lines of prior therapies of which 21 patients (48%) had prior targeted therapy, 17 patients (39%) had prior immunotherapy (IO), 23 patients (52%) had prior chemotherapy and 16 patients (36%) had prior radiation therapy.

For RAF-1i therapy, 21 patients (48%) received dabrafenib, while 10 patients (23%) received vemurafenib and encorafenib, respectively and 3 patients (7%) received investigational therapy as part of clinical trial participation. The majority of patients had an Eastern Cooperative Oncology Group (ECOG) of 1 (*n* = 30) followed by ECOG of 0 (*n* = 13) and ECOG of 2 (*n* = 1). Most patients had no CNS disease at the time of RAF-1i therapy (*n* = 36; 82%). The burden of metastatic disease sites ranged from 0 to 7 sites with at least 15 patients (34%) with 1 metastatic site at therapy. 24 patients (55%) had RAF-1i as an investigational agent while 20 patients (45%) had RAF-1i as standard of care (SOC) therapy. 37 patients (84%) received RAF-1i as part of combination therapy with immune checkpoint inhibitors (ICI) (*n* = 9; 20%), therapy targeting *MEK*, Epidermal Growth Factor Receptor (*EGFR*), B-cell lymphoma 2 (*BCL2*), and multi-kinase pathways (*n* = 34; 77%) or chemotherapy (*n* = 6; 14%).

### Safety and tolerability

Toxicities were evaluated based on the National Cancer Institute (NCI) Common Terminology Criteria for Adverse Events version 4 or 5 (CTCAE) [9]. The median treatment duration was 7 months for RAF-1i while the median number of cycles with RAF-1i was 7 (range: 1–70). Thirty-six patients came off therapy due to PD (82%) while 6 patients (14%) came off therapy due to toxicities as detailed below and 2 patients (5%) completed duration of intended therapy either as standard of care or as part of a clinical trial. Eleven patients (25%) developed grade 3 or 4 toxicities (G3 or G4) secondary to RAF-1i therapy. Of this, 6 patients developed treatment-related adverse events (TRAEs) secondary to investigational therapy such as G3 fatigue (*n* = 1), G3 neutropenia (*n* = 2), G3 transaminitis (*n* = 1) and G3 cutaneous rash (*n* = 2) where 4 patients received combination regimens with ICI, BCL-2i or MEKi. Five patients developed G3/G4 toxicities to combinatory standard of care therapies targeting *BRAF* and *MEK* pathway with G3 cutaneous rash (*n* = 3), G3 pyrexia (*n* = 1), and G3 acute kidney injury (*n* = 1). Hence, six patients discontinued therapy secondary to the above toxicities. Notably, four of these patients had the best responses with 3 partial responses (PR) and 1 complete response (CR) with a median duration of response (DOR) of 27.4 months which included cutaneous melanoma, cholangiocarcinoma, anaplastic thyroid carcinoma (ATC) and papillary thyroid carcinoma (PTC).

### Antitumor activity

The best overall response was 36.3% seen in 16 patients (3 CRs and 13 PRs) with median DOR of 11.4 months. One patient with PTC and another patient with pleomorphic xanthoastrocytoma treated with investigational therapy experienced complete response while another patient with PTC treated with SOC therapy experienced complete response as well. Of the 13 PRs, nine partial responses were as part of investigational therapy which included TNBC (*n* = 1), NSCLC (*n* = 1), CRC (*n* = 1), cholangiocarcinoma (*n* = 2), cutaneous melanoma (*n* = 2) and ATC (*n* = 1) where 8 of the 9 patients received combinatory regimen with chemotherapy, ICI, MEKi or BCL-2i. Four patients experienced partial responses as part of SOC therapy including cutaneous melanoma (*n* = 2) and CRC (*n* = 1) which included a combination with MEKi, EGFRi, and ICI. 24 patients experienced stable disease (SD) with 11 patients experiencing durable responses greater than 6 months. The disease control rate (DCR) including CR, PR, and SD >/= 6 months was 61.3% seen in 27 patients. Four patients including 2 CRC, 1 PDAC, and 1 glioblastoma experienced progressive disease (PD) via SOC and investigational therapies after a median treatment duration of 3.2 months.

### Survival outcomes

Of the 44 patients, six patients were censored due to discontinuation for toxicities. The median progression-free survival (PFS-1) with therapy with RAF-1i either as monotherapy or combination was 8.4 months. The median PFS-1 among responders was 11.4 months. The most common reason for discontinuation of therapy was progressive disease (*n* = 36) while two patients completed therapy (*n* = 2).

### Treatment between RAF-1 and RAF-2 inhibitor therapies

Twenty-seven patients (61%) underwent intervening therapies before rechallenge with RAF-2i while seventeen patients (39%) did not have intervening therapies after RAF-1i. Investigational regimens included Phosphoinositide 3-kinase (*PIK3*)i, Janus kinase (*JAK*)1i, Porcupine homolog (*PORCN*)i, ICI, oncolytic viral therapy, cytokine therapy, extracellular signal-regulated kinase (*ERK*)i, immunomodulators targeting *TLR7/8*, antibody-drug conjugate therapy, and radiation. SOC therapies included chemotherapy, anti-VEGF (Vascular Endothelial Growth Factor) agents, ICI, BRAFi, and MEKi.

The median time to RAF-2i was 3.3 months (0.03-73.7 m) from the end of RAF-1i. In terms of survival outcomes, the median PFS with intervening therapies as stated above was 3.8 months (1-20.6 m).

### Treatment with RAF-2 inhibitor

#### Patient characteristics

Predominantly 93% (*n* = 41) of patients went on to receive RAF-2i as investigational therapy where 17 patients (41%) received in combination with an agent targeting BRAF (*n* = 2), MEK (*n* = 3), ERK (*n* = 1), BCL2 (*n* = 1), EGFR (*n* = 4), cytochrome P450 3 A (CYP3A)i (*n* = 1) and multi-kinases (*n* = 7). Twenty-four patients received investigational therapy as monotherapy. Three patients received SOC therapies with RAF-2i in combination with MEKi and/or ICIs. Most patients had an ECOG of 1 (*n* = 40; 90%) while 8 patients had CNS metastases with one patient who developed CNS involvement after RAF-1i therapy. The burden of metastatic disease was higher in this group with sites ranging from 0 to 6 sites with at least 12 patients (28%) with 3 metastatic sites at therapy.

### Safety and tolerability

The median treatment duration with RAF-2i was shorter with rechallenge at 2.6 months versus 7 months for RAF-1i while the median number of cycles with RAF-2i was also shorter at 3 (1–33) compared with RAF-1i at 7 (1–70). Nine patients developed G3/G4 TRAEs with rechallenge to RAF-2i, all related to investigational therapies. Of this, 3 patients received monotherapy and developed G3 cutaneous rash (*n* = 2) and G3thromboembolic episode (*n* = 1). Six patients received combinatory therapies targeting MEK, ERK, CYP3A, multi-kinase pathway, and EGFR and developed G3 hypertension and creatinine kinase elevation (*n* = 1), G3 cutaneous rash (*n* = 2), G3 lipase elevation (*n* = 1), G3 Alanine Aminotransferase (ALT) elevation (*n* = 1) and G3 vomiting (*n* = 1). Six patients underwent dose reduction. Interestingly, one patient with ovarian serous carcinoma currently off trial and another patient with papillary thyroid carcinoma who is still experiencing partial response had a median DOR of 15.4 months.

### Antitumor activity

**Fig. 1:**
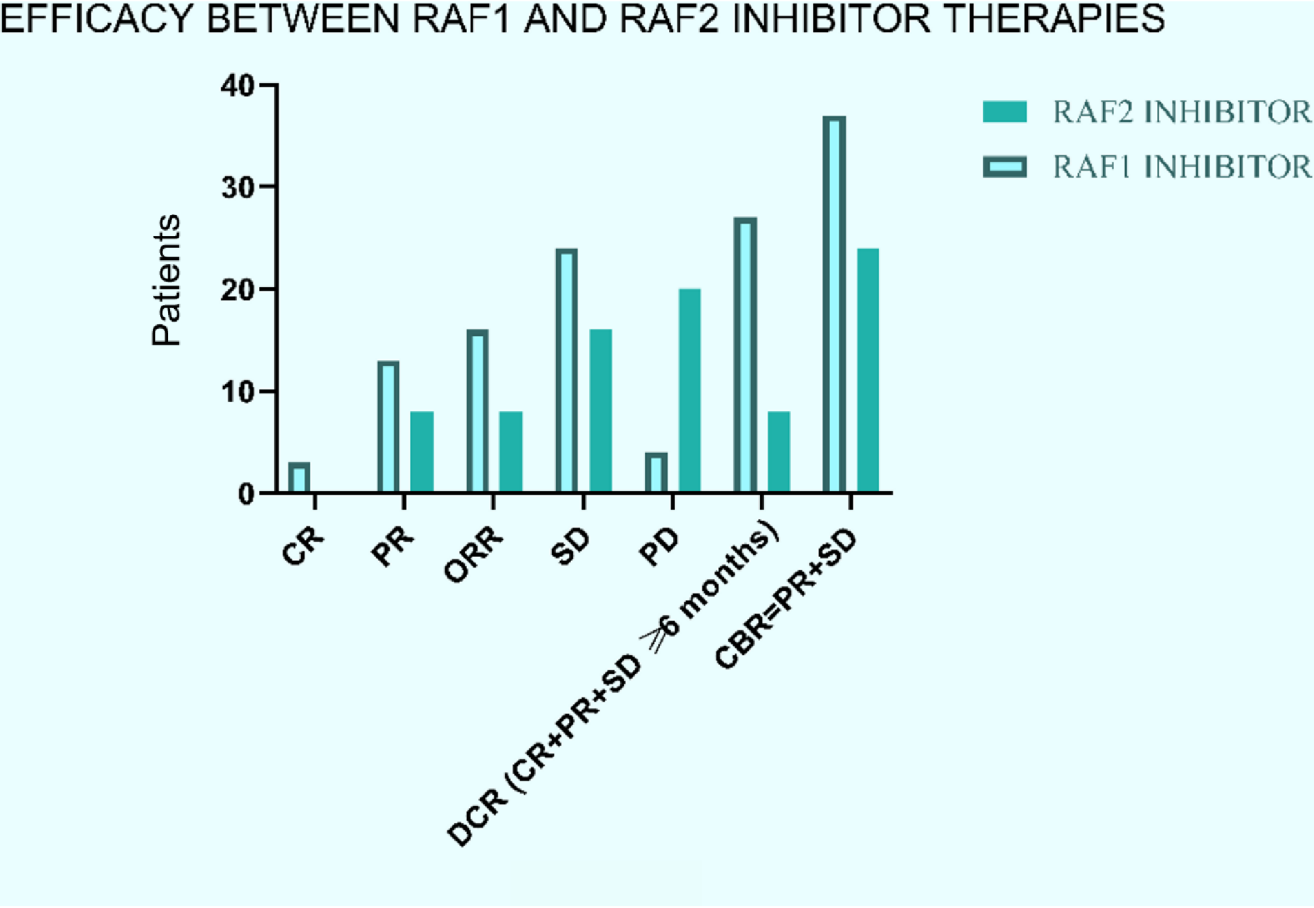
Anti-Tumor Activity of RAF2 inhibitor with rechallenge after RAF1 inhibitor. Illustration depicting the Anti-Tumor Activity of RAF2 inhibitor upon rechallenge subsequent to RAF1 inhibitor treatment. The figure visually captures the treatment response dynamics, showcasing the impact of RAF2 inhibitor rechallenge on tumor progression following initial RAF1 inhibitor therapy. CR = Complete Response; PR = Partial Response; ORR = Overall Response Rate; SD = Stable Disease; PD = Progressive Disease; DCR = Disease Control Rate; CBR = Clinical Benefit Rate; RAF1i = First RAF inhibitor; RAF2i = Second RAF inhibitor

**Fig. 2:**
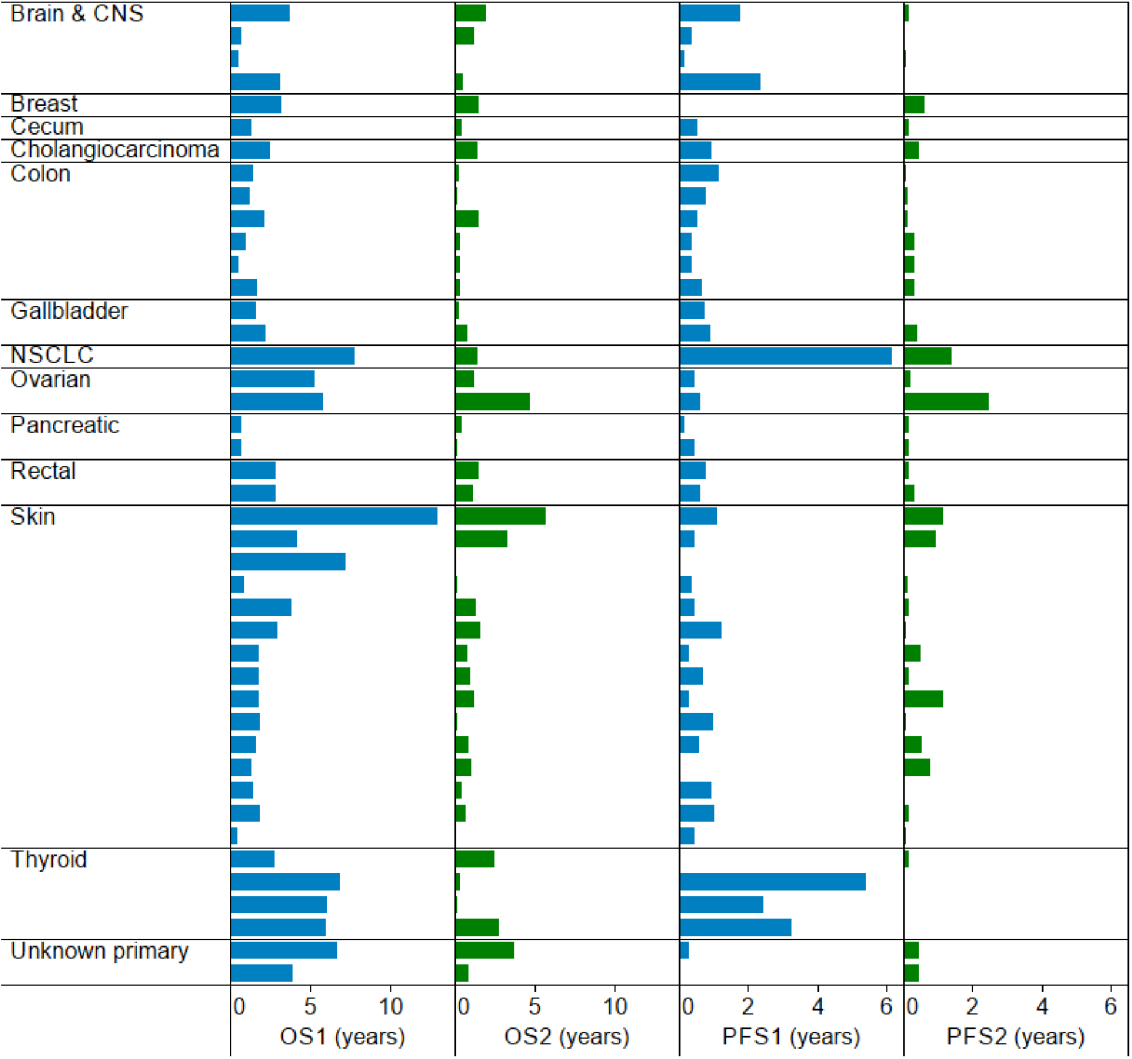
Overall Survival and Progression Free Survival Stratified by Histology in the Re-RAFFLE Study. The figure provides a comprehensive overview of treatment responses categorized by histological subtypes, highlighting the survival outcomes observed in the context of the Re-RAFFLE study. NSCLC = Non-Small Cell Lung Cancer; CRC = Colorectal Cancer; CUP = Carcinoma of Unknown Primary; OS1 = Overall Survival with first RAF inhibitor; OS2 = Overall Survival with second RAF inhibitor; PFS1 = Progression Free-Survival with first RAF inhibitor; PFS2 = Progression Free-Survival with second RAF inhibitor

The best overall response was 18.1% in eight patients to rechallenge with RAF-2i with a partial response of 18% (7 cPR + 1 uPR). Sixteen patients experienced SD (36%) while 20 patients developed PD (45%) while the clinical benefit rate (PR + SD) was greater than 50% in the participants (54.5%). Of 24 patients with PR and SD, 8 (33%) patients had durable responses (3 PRs and 5 SD) lasting greater than 6 months (Table S2). PR’s were seen in thyroid cancer (1 anaplastic; 2 papillary), 1 ovarian serous histology, 2 cutaneous melanoma, 1 cholangiocarcinoma, and 1 anaplastic astrocytoma. However, the median DOR with RAF-2i was 2.5 months compared to 11.4 months with RAF-1i. Six patients received investigational therapy while 2 patients received SOC. Of the 36 patients who discontinued RAF-1i due to PD, 17% responded to rechallenge with 6 partial responses to RAF-2i. Among 16 patients who had CR or PR on RAF1i, 5 patients experienced partial responses again with RAF-2i while 3 patients who experienced SD with RAF-1i, had conversion to PR with RAF-2i. In the RAF-2i group, of all patients achieving PR (*n* = 8), 5 patients had other intervening therapies before RAF-2i (ICI; kinase inhibitors; chemotherapy; investigational therapies) while 3 had no interim therapies. 5 responders received a combination with MEKi, while 1 responder had MEKi and ICI and 2 responders had monotherapy with RAF-2i. Notably, all responders had a median time off therapy from RAF-1i at 5.6 months. The bar plot of responses between RAF-1i and RAF-2i therapies is depicted in Fig. 1 and stratified by histology and survival impact in Fig. 2.

### Survival outcomes

Of the 44 patients, 8 patients were censored due to discontinuation for toxicities (*n* = 2), patient preference (*n* = 2), and on trial (*n* = 4). The median progression-free survival (PFS-2) with therapy with RAF-2i either as monotherapy or combination was shorter at 2.3 months (1.83-5.6 m) compared to 8.6 months (6.5-11.5 m) with RAF-1i. Of the eight rechallenge responders, four patients were censored as they remain on trial. However, the median PFS with RAF-2i responders (PFS-2) was improved at 12.8 months compared to 11.4 months with RAF-1i responders. At a median follow-up of 20 m, the median OS from retreatment with RAF-2i was 15.4 months (11.1-30.8 m). The median overall survival was 83.3 months for all patients who underwent rechallenge (39.1 m-N/A). RAF-2i therapy demonstrated a significantly inferior impact on PFS compared to RAF-1i therapy (Hazard Ratio [HR]: 2.2989, 95% confidence interval [CI]: 1.42–3.68, *p* = 0.000631). Concordantly, RAF-2i demonstrated a significant impact on OS with an HR of 2.62 (95% CI: 1.48–4.63, *p* = 0.000961). These survival graphs are depicted in Figure S1. However, one must interpret these findings with caution since this is a retrospective observational study with a small sample size.

### Prognostic factors

Independent prognostic factors for significant inferior survival outcomes at rechallenge included male gender treated with RAF-2i, metastatic burden at the time of RAF-2i, ECOG 0 and 1 with RAF-2i while rechallenge with combination RAF-2i had a worse survival impact on PFS compared to RAF-2i monotherapy (PFS: 11.6 m vs. 2.7 m; HR: 3.6; *p* = 0.037). Further characteristics are provided in the univariate analysis provided in Table S3. Specific factors that impact survival outcomes are presented in Figure S2.

Receiving intervening therapies or not did not have a significant impact on survival for RAF2i rechallenge (HR:0.6143, 95% CI: 0.2932–1.287, *p* = 0.2 [PFS]; HR:0.9674, 95% CI: 0.4215-2.21, *p* = 0.94 [OS]) as in Figure S3. ORR did not significantly differ between the combination and monotherapy groups in RAF-2i (*p* = 0.056). However, the median PFS in the patients who received intervening therapies was 3.8 months (2.37-11 m).

In terms of combination therapies, RAF-2i regimens had a significantly worse impact on overall survival compared to combination RAF-1i regimens (HR = 3.5677, *p* = 0.0159). Similarly, The RAF-2i combination showed a significant association with a worse impact on PFS (HR = 6.2360, *p* = 0.00145) as depicted in Figure S4.

When examining the impact of SOC and investigational therapies on RAF-1i and RAF-2i, investigational RAF-2i demonstrated a significantly inferior outcome on PFS with an HR of 2.8753 (*p* < 0.001) and on OS with HR of 2.9158 (*p* = 0.0019) while no significant impact was noted with SOC therapies with RAF-1i or RAF-2i (Figure S5).

### Genomic landscape

At baseline, 41 patients had *BRAF V600E* aberration, while aberrations in the RAF/RAS pathway were seen as *BRAF V600K* (*n* = 1); *BRAF K601Q* (*n* = 1) and *KRAS G12S* (*n* = 1). 13 patients had genomic testing post-RAF-1i or RAF-2i. Nine patients had a liquid biopsy and four patients had tissue based next-generation sequencing (NGS) performed on post-therapy tumor biopsies. Of the 9 patients with post-therapy liquid biopsy, 4 patients had prior tissue based somatic NGS testing. One patient with rectal adenocarcinoma had baseline *BRAF V600E* and *PIK3CA I391M* mutation at baseline tumor NGS while post-therapy liquid biopsy revealed persistence of *BRAF V600E* mutation, loss of *PIK3CA* aberration, the emergence of *APC L629*; *APC T1556fs*; *EGFR 1134delins*; *GNAS R201C* and *TP53 V203fs* aberrations with variant allele frequency (VAF) > 2% in each. Another patient with CRC had baseline tumor NGS with *BRAF V600E*; *RNF43 V36fs* and *TP53 Q167* aberrations while post-therapy liquid biopsy revealed persistence of *BRAF V600E* and *TP53 Q167* aberrations while emergent aberrations with *CDK6* gain; *EGFR* gain and *MYC* gain was noted. A cholangiocarcinoma patient with baseline *BRAF V600E* mutation and *BRAF* amplification on tumor NGS revealed the persistence of *BRAF V600E* aberration and development of *ESR1 L536F* aberration on post-therapy liquid biopsy. The last patient with ovarian serous carcinoma had a baseline *BRAF V600E* mutation via tumor NGS with the persistence of *BRAF V600E* aberration and no newly acquired aberrations on post-therapy liquid biopsy.

## Discussion

This study explored the activity of RAF inhibitors (RAFi) among diverse patients with BRAF-aberrant solid tumors beyond melanoma, who underwent rechallenge with RAF pathway-directed treatment in the context of early-phase clinical trials. Rechallenge with RAFi (s) resulted in clinical benefit rate of 54.5% with an overall response rate of 18% and a median DOR of 2.5 months. Durable responses were seen in 33% of the participants (*n* = 8/24) lasting greater than 6 months as well. Rechallenge with RAF-2i produced an improved benefit in PFS when compared with RAF-1i (12.8 months vs. 11.4 months) and median OS was extended by 10.5 months with no impact on intervening therapies or duration from rechallenge. This points to the sustainability of rechallenging the same oncogenic driver regardless of treatment breaks or other therapies.

This study presents intriguing findings on the anti-tumor activity of RAF inhibitors (RAF-1i and RAF-2i) in various solid tumors. Notable partial responses were observed in thyroid cancer, ovarian serous histology, cutaneous melanoma, cholangiocarcinoma, and anaplastic astrocytoma. However, the DOR was shorter with RAF-2i compared to RAF-1i. Rechallenge with RAF-2i showed promising responses in 14% of patients who previously discontinued RAF-1i due to disease progression. Interestingly, responders in the RAF-2i group had a median time off therapy from RAF-1i of 5.6 months although 3 patients did not have any intervening therapies, suggesting a potential impact of treatment duration on subsequent responses. While this retrospective study contributes valuable insights, it’s crucial to recognize certain limitations. The diverse inclusion of various tumor types provides a comprehensive perspective, but it may affect generalizability due to potential varied responses to RAF inhibitors across different tumor types. Limited information on treatment cycle durations, potential confounding of adverse effects by accompanying therapies, and variability in prior therapies introduce complexities in interpreting treatment responses and survival outcomes. The sample size of 44 patients, while offering valuable data, may have some constraints in statistical power. The 20-month median follow-up, while informative, might be relatively short for assessing more extended outcomes.

However, transitioning to RAF-2i therapies led to inferior survival outcomes, highlighting the importance of understanding prognostic factors to optimize treatment strategies in this patient population. Although, the median PFS-2 with rechallenge therapy with an RAF-2i either as monotherapy or combination was shorter at 2.3 months compared to 8.6 months with RAF-1i, notably this did extend the median OS from retreatment with RAF-2i by 15.4 months. Since this study involved investigational agents targeting the *RAF* pathway beyond only *BRAF* inhibition, some caveats need to be considered. Many of the RAF-2i (s) were investigational therapies in dose-finding phases and the maximum potential of anti-tumor activity could not have been reached. Despite this, notably among 54% of patients treated with investigational RAF-2i, 25% of patients were responders to rechallenge. This speaks to the potential of retargeting the RAF pathway in a tumor-agnostic fashion with an impact on anti-tumor activity and survival outcomes. Moreover, combinations with other classes of agents including chemotherapy, immunotherapy, and targeted therapies could confound tolerability, safety, and anti-tumor activity leading to a median treatment duration shorter with rechallenge at 2.6 months versus 7 months with RAF-1i. Moreover, the burden of metastatic disease was higher in this group with sites ranging from 0 to 6 sites with at least 12 patients (28%) with 3 metastatic sites at therapy.

Multiple prior studies have shown the benefit of rechallenge by retargeting the oncogenic *BRAF* driver pathway, especially melanoma. Johnson et al. rechallenged with dabrafenib and trametinib in 71 patients with advanced *BRAF V600E* mutated melanoma in an open-label phase I/II study treated previously with either dabrafenib or vemurafenib monotherapy and reported an ORR of 13-15% in two cohorts with or without crossover from dabrafenib monotherapy. Patients previously treated with dabrafenib for greater than six months demonstrated improved outcomes with the addition of MEKi compared to patients previously treated less than six months, showing a median PFS of 3.9 months versus 1.8 months (HR, 0.49; *p* = 0.02) and an ORR of 26% versus 0% [10]. In another retrospective study with advanced melanoma (*n* = 60) re-challenge with a BRAFi (BRAF2i) +/- MEKi after progression on prior BRAF inhibitor treatment (BRAF1i) was investigated. Re-challenge with BRAF2i resulted in an ORR of 28%, while the median PFS was 5.0 months, and the DOR was 14.0 months. Previous response to BRAF1i was the main predictive factor for response to BRAF2i. The addition of MEK inhibition to BRAF2i did not significantly improve outcomes compared to monotherapy or combination therapy [11]. Similarly, in another multi-institutional retrospective study with metastatic melanoma, 116 patients who previously received BRAFi with treatment-free interval, were rechallenged with BRAFi +/- MEKi. The overall response rate with BRAFi rechallenge was 43.3% while the rechallenge median OS was 9.8 months, with a median PFS of 5 months [12]. 

It should be noted not all patients respond equally, and intrinsic or acquired resistance can limit clinical efficacy especially secondary to clonal heterogeneity in tumors. Understanding the translational biology of oncogenic driver mutations and selectively choosing the apt pathways for rechallenge hold the key to practice-changing care. Resistance to BRAF/MEK inhibitors in patients with BRAF-mutant tumors can be primary or intrinsic and secondary or acquired resistance. In patients with advanced BRAF-aberrated metastatic melanoma, approximately 20% show intrinsic resistance demonstrating refractoriness to BRAF-targeted therapy [13]. This resistance is thought to arise from various mechanisms, including mutations in *RAC1*, loss of *NF1*, *NRAS* mutations, loss of *PTEN*, gain of *cyclin D1*, upregulation of *MAP3K8*, and hepatocyte growth factor [13, 14]. Acquired resistance, on the other hand, involves the recrudescence of *MAPK*-pathway signaling or other feedback loops, empowering tumor growth despite *MAPK* inhibition [15]. Significantly, the primary reason for BRAFi resistance is not the emergence of new mutations in the BRAF kinase domain that hinder the binding of the drug, which is a common phenomenon seen with other small-molecule kinase inhibitors. In contrast, resistance to BRAFi typically arises when the MAPK pathway is reactivated through various mechanisms. These mechanisms include the upregulation of receptor tyrosine kinases (RTKs) or RAS aberration, activation of downstream components such as *MEK*, *ERK*, and ULK, or development of class II or III aberrations in the BRAF gene [16, 17]. Other contributing factors may include adaptive *PI3K/AKT* signaling, changes in the expression of transcriptional regulators leading to therapy resistance, and the presence of quiescent “stem-like” cells that exhibit tolerance towards perturbations in the MAPK pathway, a phenomenon known as “drug addiction” [8]. Phenotype switching has also been postulated as a mechanism of acquired resistance, whereby melanoma cells switch cellular sensitivity with diminished dependence on the *MAPK* pathway leading to ineffective inhibition with BRAF and MEK targeted therapy [18]. This adaptability phenomenon can allow other co-occurring genetic alterations to formulate in the tumor environment to promote tumor growth despite MAPK inhibition. Additionally, resistance may arise from the development of a drug-resistant TME, allowing tumor growth due to robust extracellular matrix reorganization. Given the heterogeneity of tumors, varying genetic and epigenetic mechanisms likely contribute to BRAF/MEK resistance [15]. The concept of drug dependency in drug-resistant cells suggests that altering dosing strategies may prevent the emergence of resistance. Research has demonstrated that cessation of treatment and transitioning to an alternative therapy can result in the regression of tumors that are resistant to BRAFi. This transition may promote the emergence of rapidly proliferating clones that maintain their susceptibility to rechallenging with BRAF/MEKi [8]. 

Multiple preclinical studies have explored temporary or reversible resistance to BRAF inhibitors and the concept of a ‘plastic’ tumor phenotype. The role of TME changes and therapeutic escape mechanisms in melanoma cells may impact treatment response and resistance. Das Thakur et al. demonstrated that melanoma cells display temporary resistance to BRAF inhibitors due to their ability to adapt in response to treatment and switch between different cellular states such as proliferative and invasive phenotypes, in response to treatment pressure. Melanoma cells can activate alternative survival pathways, develop drug-tolerant subpopulations, and exhibit a “plastic” tumor phenotype. This phenotype switching may contribute to treatment resistance by enabling melanoma cells to acquire additional genetic alterations and promote survival in the presence of BRAF inhibitors. The findings highlight the importance of exploring combination therapies targeting both intrinsic and immunological factors to overcome resistance and improve treatment outcomes in *BRAF*-mutant cancers [8]. 

The plastic phenotype observed in melanoma cells upon BRAF inhibitor withdrawal indicates the dynamic nature of tumor response and potential opportunities for rechallenge in various arrays of treatment schedules [8]. In a translational study examining intermittent treatment with the BRAFV600E inhibitor, LGX818/encorafenib, was more effective in suppressing growth in human melanoma cells expressing *BRAFV600E*, *p61-BRAFV600E*, or *MEK2C125* oncogenes compared to continuous treatment. The advantageous effect of intermittent treatment appeared to be driven by re-sensitization during drug removal, followed by cell death upon drug re-addition, rather than drug addiction. The intermittent treatment also resulted in a distinct transcriptome, including mediators of cell invasiveness and the epithelial-to-mesenchymal transition, indicating phenotypic plasticity and drug re-sensitization as underlying mechanisms [19]. However, phase 2 trials employing intermittent dosing with combinations of BRAF and MEK inhibitors showed poorer PFS and no significant difference in OS compared to continuous treatment [20, 21]. Further elucidation of the mechanisms governing the response to intermittent therapy, other dosing schedules, and translation into clinical effectiveness is needed.

To overcome acquired resistance from BRAF class II and III alterations and other RAF aberrations, developing novel therapies is crucial. The investigational drug tovorafenib, a selective pan-*RAF* inhibitor, has shown clinically meaningful responses in pediatric patients with BRAF-altered low-grade gliomas (LGGs) in phase 1B and phase 2 trials. In the phase 2 FIREFLY-1 trial (NCT04775485), tovorafenib demonstrated an ORR of 64% among 77 patients, where more than 60% of patients had prior *MAPK*-targeted therapies. Responses were observed in both *BRAF* fusion/rearrangement and *V600E* mutation tumors, including those previously treated with MAPK inhibitors. The most common treatment-related adverse events were manageable and included hair color changes, increased creatine phosphokinase, anemia, fatigue, and maculopapular rash [22]. FORE8394 is another investigational inhibitor targeting class 1 (V600) and class 2 (activating non-V600) *BRAF*-altered tumors. In a phase 1/2a study, 110 patients with advanced solid or CNS tumors carrying *BRAF* alterations received FORE8394 where 25% had prior *MAPK*-targeted therapy. ORR in patients with MAPKi-naïve, V600 mutant tumors, was 39% while rechallenge in patients with V600 mutated tumors previously treated with MAPKi, ORR was 18%. The most commonly reported treatment-emergent adverse events (TEAEs) were grade 1–2 increased ALT (39%), increased AST (35%), and fatigue (34%) [23]. 

## Conclusions

RAF inhibitors have demonstrated promising antitumor activity and derived clinical benefit in patient outcomes in the rechallenge of patients with *RAF* aberrated advanced solid tumors. However, acquired resistance remains a significant challenge. Research efforts to identify resistance mechanisms, develop next-generation therapies, and optimize treatment strategies are critical for achieving long-lasting and durable responses. However, further analysis and considerations, such as sample size, clinical context, and other factors, are necessary to draw definitive conclusions about the impact of RAF-1i and RAF-2i therapies on clinical outcomes. Further prospective studies are warranted to validate these findings and expand re-challenging targeted therapy options in tumor-agnostic *BRAF*-aberrant cancers.

### Electronic supplementary material

Below is the link to the electronic supplementary material.

Supplementary Material 1

### Electronic supplementary material

Below is the link to the electronic supplementary material.

Supplementary Material 2

## Data Availability

Data availability is not openly available as it is contingent on completion of on-going studies and data analysis. Data are located in controlled access data storage at MD Anderson Cancer Center. The findings, including relevant scientific data, will be made accessible through appropriate channels or publications to facilitate further research.

## Abbreviations

RAF: Rapidly Accelerated Fibrosarcoma
BRAF: v-RAF murine sarcoma viral oncogene homolog B1
CR 1/2/3: Conserved Region
MEK: Mitogen-Activated Protein Kinase
ERK: Extracellular Signal-Regulated Protein Kinase
PI3K: Phosphoinositide 3-Kinase
RAS: Rat Sarcoma
CR: Completed Response
FDA: Food and Drug Administration
PR: Partial Response
ORR: Overall Response Rate
DOR: Duration of Response
SD: Stable Disease
PD: Progressive Disease
PFS (1/2): Progression-Free Survival
OS: Overall Survival
ECOG: Eastern Cooperative Oncology Group
SOC: Standard of Care
INV: Investigational
ICI: Immune Checkpoint Inhibitor
DCR: Disease Control Rate
HR: Hazard Ratio
CI: Confidence Interval
CYP3A: cytochrome P450 3 A
ALT: Alanine Aminotransferase
TME: Tumor Microenvironment
AKT: Protein kinase B
NRAS: Neuroblastoma RAS viral oncogene homolog
CLIA: Clinical Laboratory Improvement Amendments
CRC: Colorectal Carcinoma
CNS: Central Nervous System
EGFR: Epidermal Growth Factor Receptor
PDAC: Pancreatic Ductal Adenocarcinoma
ICI: Immune Checkpoint Inhibitors
TNBC: Triple Negative Breast Cancer
NSCLC: Non-Small Cell Lung Cancer
BCL2: B-cell lymphoma 2
VAF: Variant Allele Frequency
TRAE: Treatment Related Adverse Event
G: Grade
ATC: Anaplastic Thyroid Carcinoma
PTC: Papillary Thyroid Carcinoma
IO: Immunotherapy
VEGF: Vascular Endothelial Growth Factor
PIK3: Phosphoinositide 3-kinase
JAK: Janus kinase
PORCN: Porcupine homolog
ALT: Alanine Aminotransferase
VAF: Variant Allele Frequency
LGG: Low-Grade Gliomas
NGS: Next-Generation Sequencing
RAFi (1/2): RAF inhibitors
TEAE: Treatment-Emergent Adverse Events
