# Supplementary material for: RAF inhibitor re-challenge therapy in BRAF-aberrant pan-cancers: the RE-RAFFLE study
Source: Mol Cancer. 2024 Mar 26;23:64. doi: 10.1186/s12943-024-01982-4 (PMC10964523; doi:10.1186/s12943-024-01982-4)
Supplement: Supplementary file 1 — Supplementary Material 1 [file 12943_2024_1982_MOESM1_ESM.docx]

**Supplementary Materials and Methods**

This was a retrospective study conducted in a single institution approved by The University of Texas MD Anderson Cancer Center’s Institutional Review Board. All patients treated as part of the clinical trial provided written informed consent. The demographic, clinical, and histopathologic data of patients were retrospectively collected and analyzed. Trial designs, schedules, and assessments varied among the clinical trials involved.

*Statistical Analysis*

Demographic and clinical characteristics were analyzed using descriptive statistics. PFS was defined as the time from the first day of cycle 1 to the date of progression or death, whichever came first. Patients who were alive and progression-free at the last clinical follow-up were censored at the date of the last clinical follow-up. OS was defined as the time from the first day of cycle 1 to death from any cause. Patients alive at the last follow-up were censored at the date of the last contact. Survival (PFS and OS) were analyzed using the Kaplan-Meier method from the time of trial participation and included median survivals (with 95% CIs). HRs and corresponding CIs and P values were computed using a Cox proportional hazards regression analysis. Clopper-Pearson exact binomial CIs were provided for estimates of proportions. Survival differences between treatment cohorts were assessed through the log-rank test with univariate analysis. All tests were 2-sided, and P values < .05 were considered statistically significant. All statistical analyses were performed using R software, v3.6.0.

*Genomic Analysis*

Archived tumor specimens were analyzed at institutional Clinical Laboratory Improvement Amendments (CLIA)-certified laboratories for next-generation sequencing data. Data was reviewed using other platforms such as NeoGenomics, Aliso Viejo, CA, USA; Guardant360; Guardant Health, Redwood City, CA; and Foundation Medicine, Cambridge, MA, USA.
