# Supplementary material for: RAF inhibitor re-challenge therapy in BRAF-aberrant pan-cancers: the RE-RAFFLE study
Source: Mol Cancer. 2024 Mar 26;23:64. doi: 10.1186/s12943-024-01982-4 (PMC10964523; doi:10.1186/s12943-024-01982-4)
Supplement: Supplementary file 2 — Supplementary Material 2 [file 12943_2024_1982_MOESM2_ESM.docx]

**SUPPLEMENTARY FIGURES**

**Supplementary Fig. 1:** Overall survival and progression-free survival outcomes based on RAF1i and RAF2i therapies.

RAF1i = First RAF inhibitor; RAF2i = Second RAF inhibitor


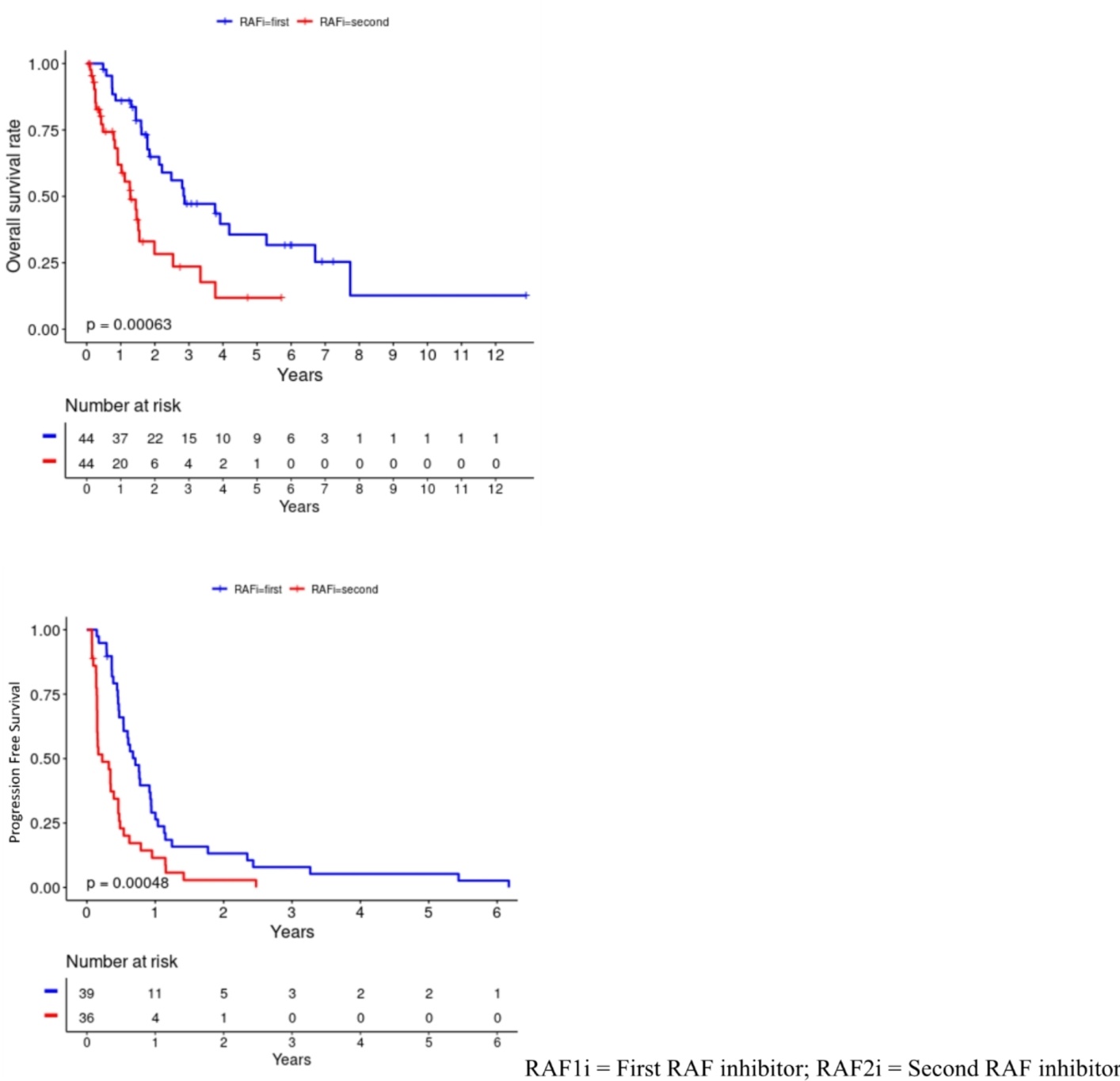


**Supplementary Fig. 2 (A; B; C):** Prognostic impact of metastatic burden, gender, ECOG on OS and PFS.

**2A.** Impact of metastatic burden of disease (0–2 lesions versus 3–5 lesions) at presentation with first RAF inhibitor and second RAF inhibitor


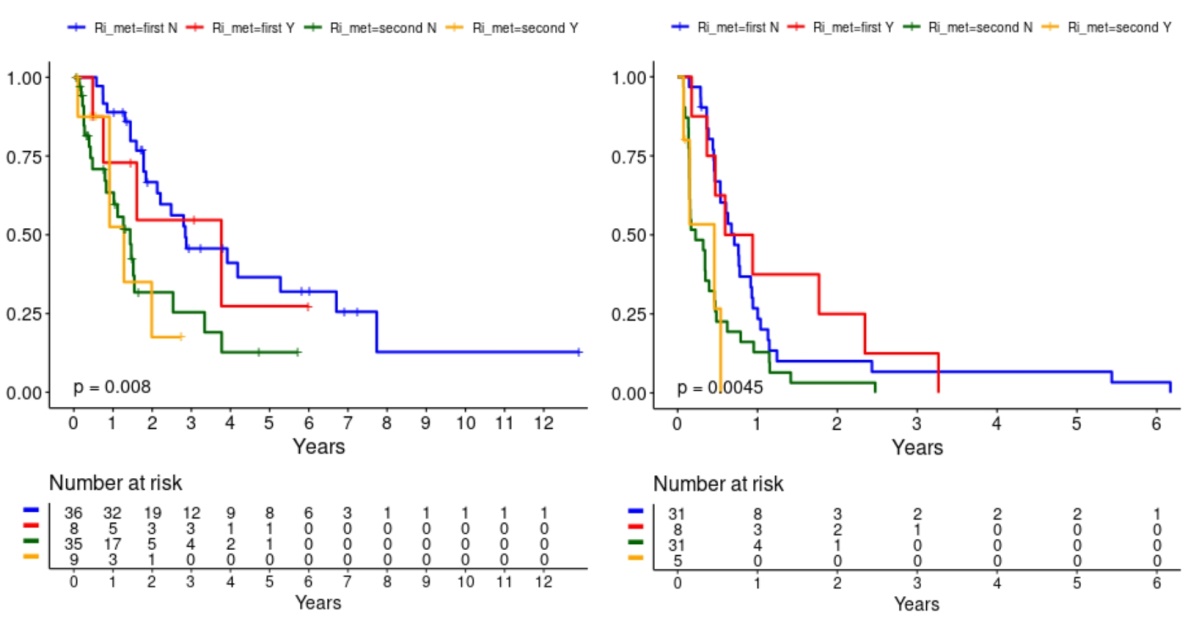


Overall Survival Progression Free Survival

**2B.** Impact of gender at presentation with first RAF inhibitor and second RAF inhibitor


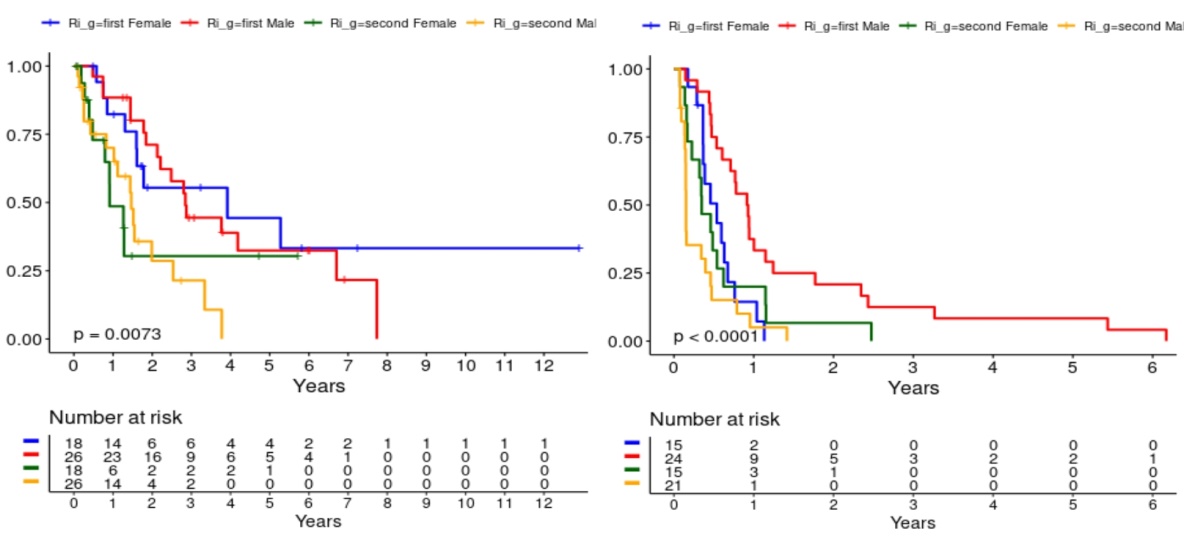


Overall Survival Progression Free Survival

**2C.** Impact of Performance Status with first RAF inhibitor and second RAF inhibitor


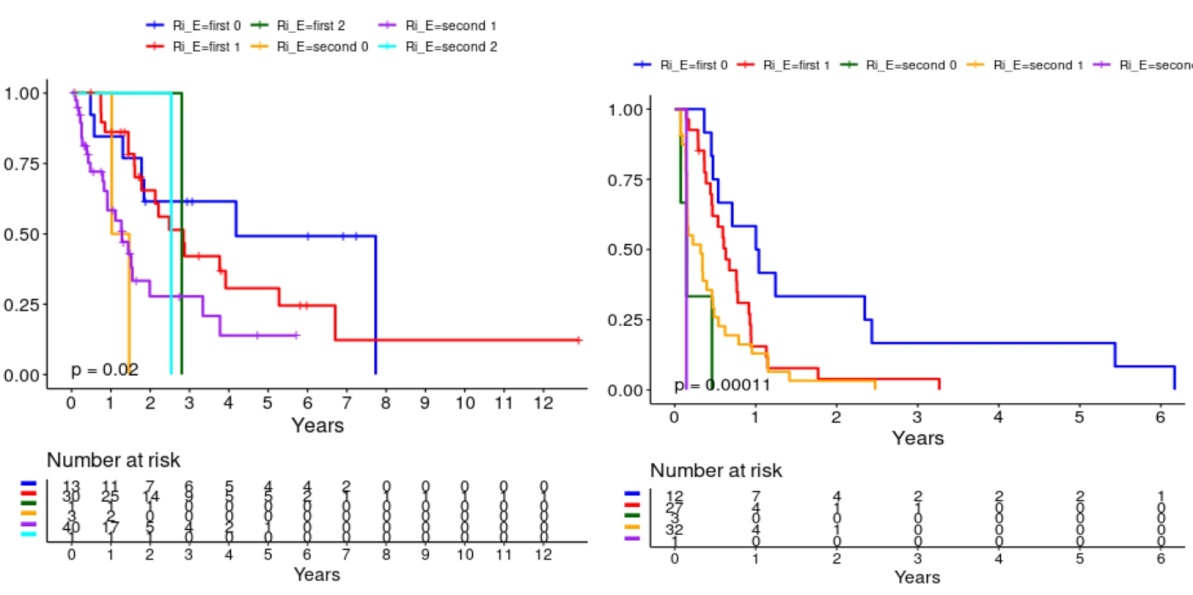


Overall Survival Progression Free Survival

**Supplementary Fig. 3:** Intervening therapies between RAF1i and RAF2i and impact on OS and PFS


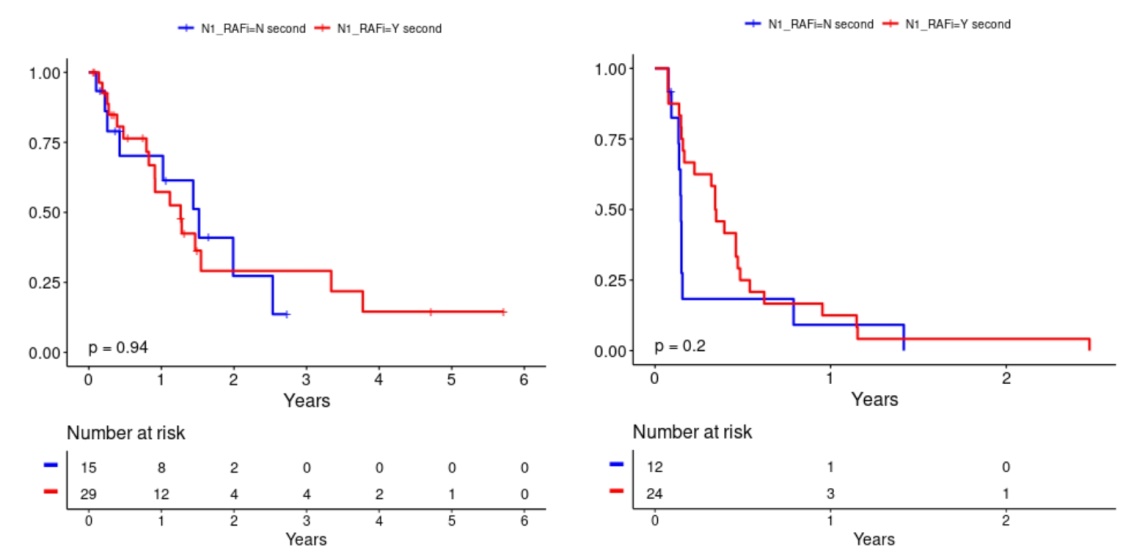


Overall Survival Progression Free Survival

**Supplementary Fig. 4:** RAF1i and RAF2i with combinations and impact on OS and PFS


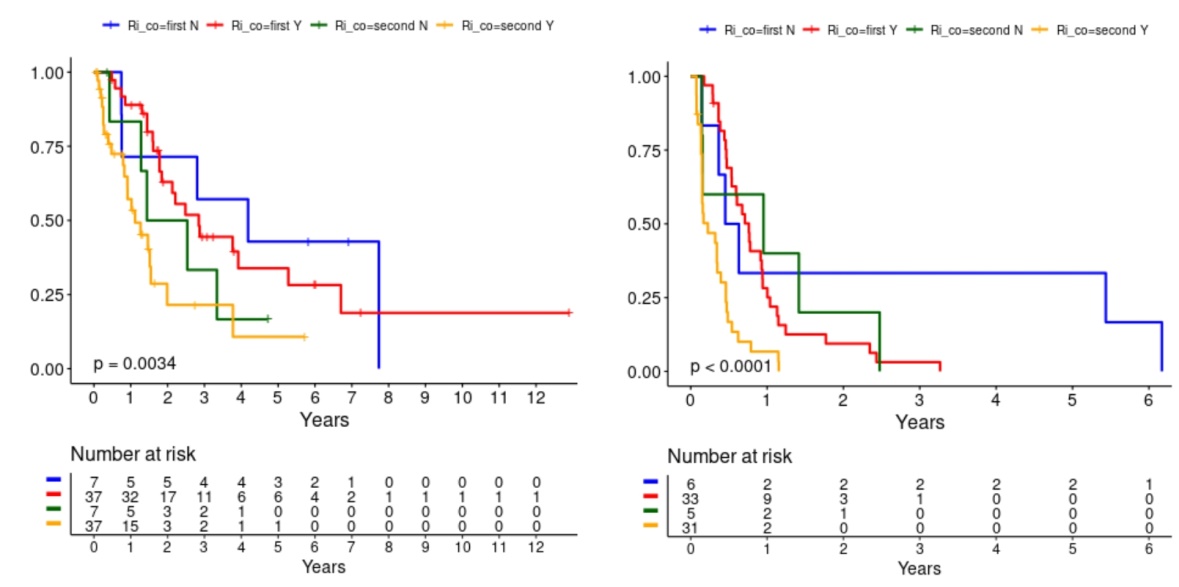


Overall Survival Progression Free Survival

**Supplementary Fig. 5:** RAF1i and RAF2i as standard of care or investigational therapies and impact on OS and PFS


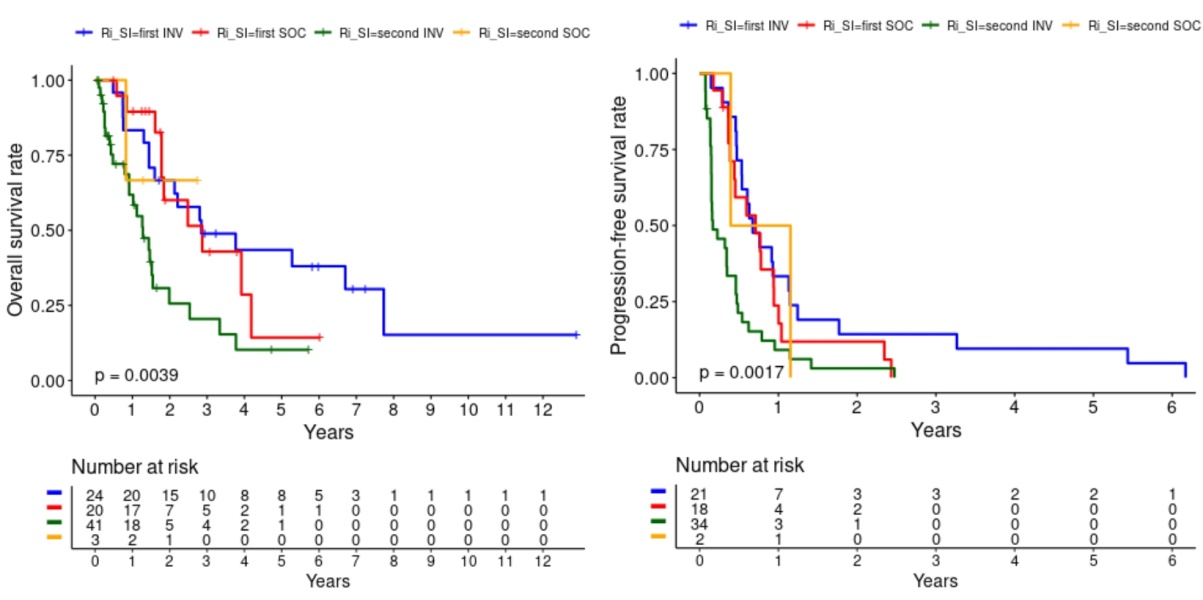


Overall Survival Progression Free Survival

**SUPPLEMENTARY TABLES**

**Supplementary Table S1.** Baseline Characteristics

| **DEMOGRAPHICS** | | **ALL PATIENTS**  **(n = 44)** |
| --- | --- | --- |
| **Age (Median Range)** | | 54.5 (25–76) |
| **Gender, n (%)** | |  |
| **Male** | | 26 (59%) |
| **Female** | | 18 (41%) |
| **Race, n (%)** | |  |
| **Caucasian** | | 36 (82%) |
| **Hispanic** | | 6 (14%) |
| **Black** | | 1 (2%) |
| **Asian** | | 1 (2%) |
| **Primary tumor, n (%)** | |  |
| **Cutaneous Melanoma** | | 16 (36%) |
| **Colorectal Adenocarcinoma** | | 10 (23%) |
| **Thyroid** | **Papillary Thyroid Carcinoma** | 3 (7%) |
|  | **Anaplastic Thyroid Cancer** | 1 (2%) |
| **Brain** | **Glioblastoma** | 2 (5%) |
|  | **Pleomorphic Xanthoastrocytoma** | 1 (2%) |
|  | **Anaplastic Astrocytoma** | 1 (2%) |
| **Cholangiocarcinoma** | | 3 (7%) |
| **Pancreatic Adenocarcinoma** | | 2 (5%) |
| **Ovarian Serous Carcinoma** | | 2 (5%) |
| **Lung Adenocarcinoma** | | 1 (2%) |
| **Triple Negative Breast Cancer** | | 1 (2%) |
| **Neuroendocrine Carcinoma** | | 1 (2%) |
| **Therapies: First RAF inhibitor** | |  |
| **Dabrafenib** | | 21 |
| **Vemurafenib** | | 10 |
| **Encorafenib** | | 10 |
| **Investigational Agents** | | 3 |
| **Therapies: Second RAF inhibitor** | |  |
| **Investigational Agents** | | 31 |
| **Dabrafenib** | | 1 |
| **Vemurafenib** | | 7 |
| **Encorafenib** | | 5 |
| **Prior lines of systemic therapies, n (%)** | |  |
| **0–1** | | 22 (%) |
| **2–3** | | 16 (%) |
| **4–5** | | 6 (%) |
| **Survival Status** | |  |
| **Alive** | | 18 (%) |
| **Dead** | | 26 (%) |
|  | |  |

RAF = Rapidly Accelerated Fibrosarcoma; n = Number

**Supplementary Table S2.** Overall Responses with First RAFi and Rechallenge with Second RAFi

| **OVERALL RESPONSES** | **RAF1 INHIBITOR (*n* = 44)** | **RAF2 INHIBITOR (*n* = 44)** |
| --- | --- | --- |
| **CR** | 3 (7%) | 0 (0%) |
| **PR** | 13 (29.5%) | 8 (18.1%) |
| **ORR** | 16 (36%) | 8 (18.1%) |
| **SD** | 24 (54.5%) | 16 (36.3%) |
| **PD** | 4 (9%) | 20 (45.4%) |
| **DCR (CR + PR + SD ≥ 6 months)** | 27 (61.3%) | 8 (18.1%) |
| **CBR = PR + SD** | 37 (84%) | 24 (54.5%) |

CR = Complete Response; PR = Partial Response; ORR = Overall Response Rate; SD = Stable Disease; PD = Progressive Disease.

DCR = Disease Control Rate; CBR = Clinical Benefit Rate; RAFi = Rapidly Accelerated Fibrosarcoma Inhibitor

**Supplementary Table S3.** Univariate Analysis of Patient Characteristics and Survival Outcomes

|  | | | | | |
| --- | --- | --- | --- | --- | --- |
| **CHARACTERISTIC** | | **OS HR (95% CI)** | **P VALUE** | **PFS HR (95% CI)** | **P VALUE** |
| **Age** | | 0.993 (0.965–1.02) | 0.615 | 0.982 (0.960-1.00) | 0.0976 |
| **Gender: Male** | | 3.05 (1.32–7.04) | 0.00894 | 0.442 (0.218–0.897) | 0.0238 |
| **Tumor type**  Skin  Brain & CNS  Breast  Cecum  Cholangiocarcinoma  Colon  Gallbladder  NSCLC  Ovarian  Pancreatic  Rectal  Thyroid  Unknown Primary | | Reference  1.62 (0.315–8.35)  4.35E-08 (0-Inf)  18.6 (1.73-201.107)  2.85 (0.320-25.418)  6.25 (1.49–26.3)  4.90 (0.894–26.9)  0.470 (0.0539-4.09)  0.427 (0.0504-3.62)  58.9 (6.34–548)  1.96 (0.374–10.3)  0.238 (0.0286-1.98)  0.890 (0.180–4.41) | 0.564  0.998  0.0161  0.348  0.0124  0.0672  0.494  0.435  0.000339  0.426  0.184  0.886 | Reference  0.32 (0.0710–1.44)  1 (1–1)  2.41 (0.315–18.5)  0.691 (0.0921-5.19)  1.25 (0.532–2.92)  1.03 (0.240–4.43)  5.56E-17 (0-Inf)  2.41 (0.553–10.5)  6.54 (1.42-30.0)  1.37 (0.319–5.88)  2.97E-09 (0-Inf)  14.5 (1.502-139) | 0.138  NA  0.397  0.719  0.611  0.967  0.995  0.241  0.0157  0.672  0.995  0.0208 |
|  |  |  |  |  |  |
| **ECOG: 0–1** | | 3.529 (1.46–8.54) | 0.00514 | 3.510 (1.69–7.30) | 0.000779 |
| **Ethnicity** | **White** | Reference |  |  |  |
|  | **Asian** | 1.26E-08 (0-Inf) | 0.998 | 0.259 (0.0334-2.01) | 0.196 |
|  | **Black** | 1.43 (0.188–10.8) | 0.731 | 1.637 (0.216–12.41) | 0.634 |
|  | **Hispanic** | 0.701 (0.207–2.38) | 0.568 | 0.893 (0.339–2.35) | 0.819 |
|  | **Native American** | N/A |  |  |  |
| **Metastatic sites (0–2 lesions)** | | Reference |  |  |  |
| **Metastatic sites (3–5 lesions)** | | 1.64 (0.722–3.72) | 0.238 | 2.75 (1.24–6.11) | 0.0127 |
| **RAF1i vs RAF2i** | | 2.62 (1.48–4.63) | 0.000961 | 2.29 (1.42–3.68) | 0.000631 |
| **SOC vs INV therapies** | | 2.916 (1.48–5.73) | 0.0019 | 2.88 (1.61–5.15) | 0.000384 |
| **RAFi combination vs monotherapy** | | 3.57 (1.27-10.0) | 0.0159 | 6.24 (2.02–19.2) | 0.00145 |
| **Presence of brain metastases** | | 1.22 (0.417–3.59) | 0.713 | 0.838 (0.3776–1.858) | 0.663 |
| **Combination with IO** | | 1.05 (0.391–2.84) | 0.919 | 1.09 (0.488–2.44) | 0.833 |
| **Combination with TT** | | 2.34 (0.790–6.92) | 0.125 | 2.33 (0.882–6.13) | 0.088 |
| **Combination with CT** | | 3.82 (1.16–12.5) | 0.0274 | 1.38 (0.561–3.37) | 0.486 |

OS = Overall Survival; PFS = Progression Free Survival; HR = Hazard Ratio; CI = Confidence Interval; inf = infimum; CNS = Central Nervous System; NA = Not Available; NSCLC = Non-Small Cell Lung Cancer; ECOG = Eastern Cooperative Oncology Group; RAF1i = First RAF inhibitor; RAF2i = Second RAF inhibitor; SOC = Standard of Care; INV = Investigational; IO = Immunotherapy; TT = Targeted Therapy; CT = Chemotherapy
